# Classification of non-small cell lung cancer by histologic subtype using deep learning in public and private data sets of computed tomography images

**DOI:** 10.1590/0100-3984.2024.0093

**Published:** 2025-05-20

**Authors:** Marcos Antonio Dias Lima, Carlos Frederico Motta Vasconcelos, Roberto Macoto Ichinose, Antonio Mauricio Ferreira Leite Miranda de Sá

**Affiliations:** 1 Instituto Alberto Luiz Coimbra de Pós-Graduação e Pesquisa de Engenharia – Universidade Federal do Rio de Janeiro (COPPE-UFRJ), Rio de Janeiro, RJ, Brazil; 2 Instituto Nacional de Câncer (INCA), Rio de Janeiro, RJ, Brazil

**Keywords:** Carcinoma, non-small-cell lung, Image processing, computer-assisted/methods, Segmentation, Semantics, Carcinoma/classification, Deep learning, Carcinoma pulmonar de células não pequenas, Processamento de imagem assistida por computador, Segmentação, Semântica, Carcinoma/classificação, Aprendizagem profunda

## Abstract

**Objective:**

To develop a deep learning system to classify non-small cell lung cancer (NSCLC) by histologic subtype—adenocarcinoma or squamous cell carcinoma (SCC)—from computed tomography (CT) images in which the tumor regions were segmented, comparing our results with those of similar studies conducted in other countries and evaluating the accuracy of automated classification by using data from the Instituto Nacional de Câncer, Brazil.

**Materials and Methods:**

To develop the classification system, we employed a 2D U-Net neural network for semantic segmentation, with data augmentation and preprocessing steps. It was pretrained on 28,506 CT images from The Cancer Image Archive, a private database, and validated on 2,015 of those images. To develop the classification algorithm, we used a VGG16-based network, modified for better performance, with 3,080 images of adenocarcinoma and SCC from the Instituto Nacional de Câncer database.

**Results:**

The algorithm achieved an accuracy of 84.5% for detecting adenocarcinoma and 89.6% for detecting SCC, with sensitivities of 91.7% and 90.4%, respectively, which are considered satisfactory when compared with the values obtained in similar studies.

**Conclusion:**

The system developed appears to provide accurate automated detection, as well as tumor segmentation and classification of NSCLC subtypes of a local population using deep learning networks trained using public image data sets. This method could assist oncological radiologists by improving the efficiency of preliminary diagnoses

## Abstract (pt)

**Objetivo:**

Desenvolver um sistema baseado em técnicas de aprendizado profundo para classificação dos principais subtipos histológicos de câncer de pulmão não pequenas células (CPNPC) – adenocarcinoma e carcinoma de células escamosas (CCE) – usando imagens tomográficas. O sistema desenvolvido segmenta o tumor nas imagens, classificando as lesões em adenocarcinoma e CCE. Comparamos os resultados com estudos similares e avaliamos a precisão na classificação automática dos subtipos de CPNPC utilizando imagens fornecidas pelo Instituto Nacional de Câncer.

**Materiais e Métodos:**

A rede usada para segmentação semântica foi baseada na arquitetura 2D U-Net com etapa de pré-processamento utilizando técnicas de aumento de dados. Esta rede foi pré-treinada com 28.506 imagens e validada com 2.015 imagens usando base de dados de um repositório público de informações de imagens conhecido internacionalmente como The Cancer Image Archive. Para classificação foi utilizada rede baseada na arquitetura VGG16, modificada para melhorar o desempenho, usando 3.080 imagens de adenocarcinoma e CCE do Instituto Nacional de Câncer.

**Resultados:**

O sistema fornece detecção da lesão com precisão de 84,5% para adenocarcinoma e 89,6 para CCE, com sensibilidade de 91,7% e 90,4%, respectivamente, considerada adequada quando comparada com estudos semelhantes.

**Conclusão:**

Este sistema oferece um método preciso de detecção, segmentação e classificação para um conjunto reduzido de pacientes, que pode ajudar radiologistas na análise de imagens e acelerar o diagnóstico preliminar.

## INTRODUCTION

Early cancer detection influences the disease outcome^**(^1^)**^. That motivated the present study, which aims to aid in the classification of the predominant histologic subtypes of lung cancer. Lung cancer is a major public health issue in Brazil. According to the Brazilian Instituto Nacional de Câncer (INCA, National Cancer Institute), a combined 32,560 cases of lung, trachea, and bronchial cancer were expected in 2024^**(^2^)**^. Non-small cell lung cancer (NSCLC) is the most common type, accounting for 85% of all lung cancer cases. There are two main subtypes of NSCLC: adenocarcinoma and squamous cell carcinoma (SCC). In 60% of cases, NSCLC is detected at an advanced stage, resulting in a global 5-year survival rate of only 10–15%^**(^3^,^4^)**^. Biopsy, albeit essential for confirming malignancy, has limitations due to the small size of the specimens and therefore might may not fully capture the heterogeneity of a given tumor. Accurate delineation of the tumor is a crucial first step in its classification^**(^5^)**^. Because stable, accurate segmentation is critical, an automated, reproducible lung tumor delineation algorithm would facilitate that classification. In addition, some images cannot be segmented in only one step, and additional steps are therefore required; in many cases, the radiologist must scroll through many computed tomography (CT) slices to determine what part of the segmentation is missing^**(^6^)**^. Medical image segmentation generally consists of two related tasks: object recognition and object delineation. Accurate image segmentation methods are vital for proper disease detection, histologic classification of tumors, diagnosis, treatment planning, and follow-up. In addition to the conventional region-of-interest analysis, an accurate image segmentation method is often needed for diagnostic or prognostic assessment^**(^7^–^10^)**^.

### Deep learning applications

Deep learning (DL) techniques have been increasingly used in order to address challenges in the detection and classification of tumors, requiring large annotated data sets for effective learning^**(^11^)**^. Recent advancements include hybrid regional networks for automated tumor segmentation from positron-emission tomography (PET)/CT images^**(^12^)**^, fully automated pipelines for volumetric segmentation of NSCLC^**(^13^)**^, and computer-aided diagnostic methods for CT images^**(^14^)**^. The World Health Organization emphasizes the importance of staging and histologic classification for treatment and prognosis^**(^15^)**^. Networks employing DL could enhance the classification of NSCLC subtypes by leveraging various image data sets, tailored for both public and local populations.

## MATERIALS AND METHODS

This work was approved by the INCA Board of Ethics (Reference no. 6.331.223; 14/09/2022). We analyzed 2,005 examinations in the INCA database with the predominant histologic subtypes, of which 1,172 (58.5%) were adenocarcinoma and 833 (41.5%) were SCC. The criteria for patient selection were as follows: having a tumor classified as stage I, II or III; with having a tumor for which the largest diameter was ≤ 7 cm; having no metastases; and CT image acquisition and classification of the histologic subtype having occurred within two weeks of each other. From the INCA database, we selected 104 patients to use for the validation—62 with adenocarcinomas and 42 with SCCs—which reflects a ratio between the subtypes similar to that of the sample as a whole. For the classification, we selected 62 INCA patients: 30 with adenocarcinomas and 32 with SCCs. For validation, we used 81 patients registered in The Cancer Image Archive (TCIA): 41 with adenocarcinomas and 40 with SCCs. Thus, we maintained the balance of the data sets for each subtype.

The pipeline developed for this research uses two convolutional neural networks (CNNs) written in Python, version 3.9, using the Tensor Flow and Keras frameworks for model implementation and the scikit-learn library for the evaluation model. The first CNN employs a 2D U-Net architecture^**(^16^)**^ that uses convolutional layers to detect local features in input images. Each convolutional layer connects to a small subset of spatially connected neurons, with shared connection weights enhancing the detection of local structures. This architecture includes pooling layers to reduce computational complexity and extract hierarchical image features. The second CNN is based on the VGG16 model proposed by Simonyan & Zisserman^**(^17^)**^. This model, noted for its high performance on ImageNet^**(^18^,^19^)**^, features a deep network with 13 convolutional layers, five max-pooling layers, and three dense layers. The VGG16 design includes smaller filters and increased depth and filter count after each max-pooling layer. Transfer learning is used to adjust the final layers for improved classification of adenocarcinoma and SCC. The DL algorithm was developed in Python using the Keras API and TensorFlow framework; all tasks were performed on a system with an 8th generation Intel i7 processor, 16 GB RAM, and a NVIDIA GeForce GTX 1060 graphics processing unit (GPU).

### Data set

For the purposes of this study, we employed TCIA^**(^20^)**^, a public database which, at our access, contained 442 scans of patients with NSCLC, including clinical annotations for radiomics. This database also provides the ground truth segmentation images of the NSCLC tumors, defined by a specialist board, representing the tumor label with the same shape as the corresponding CT image. For training, 122 scans were selected from the TCIA data set and categorized as adenocarcinoma, SCC, or “not otherwise specified”. For external validation, we used 104 PET/CT scans from INCA patients diagnosed with NSCLC between 2016 and 2020. These scans averaged 600 slices each, from a Philips Gemini TF PET/CT scanner with standardized scanning protocols, with a tube voltage of 120 kVp, a tube current of 213 mAs, mediastinal window settings, and a slice thickness of 1 mm, without contrast.

### Preprocessing

The preprocessing steps aimed to optimize data for training^**(^21^)**^ and conform to GPU memory limitations. Original images (512 × 512 pixels) were resized (to 256 × 256 pixels) to retain quality while ensuring sufficient spatial resolution. The preprocessing consisted of isolating the lung regions by morphological effects (erosion and dilation) after applying a threshold. A mask for the lung region only was then applied to the original image, and a masked image was applied to the input network. This procedure was also used on the INCA image data set during the validation.

### Data augmentation

The TCIA data set initially contained 10,025 images, of which only 1,057 contained NSCLC information. Geometric transformations were applied to address this imbalance and enhance training. Horizontal mirroring increased the data set to 20,050 samples. Additional augmentations^**(^22^)**^, including rotations (10° and −10°) and elastic deformations (random displacement fields), further expanded the data set to 28,506 images. This same data augmentation sequence was used on the INCA database images only in the training stage of the classification network.

### Metrics

To evaluate the results from the DL algorithm, some metrics were calculated. For the validation testing of the segmentation results using the TCIA database, we calculated the Dice similarity coefficient (DSC), a statistical index that evaluates the similarity between two sets of data and has become one of the most widely used tools in the validation of image segmentation algorithms^**(^20^)**^, as follows:


Eq. 1 $$DSC=\frac{2\left|X\cap Y\right|}{\left|X\right|+\left|Y\right|}$$

where |*X*| is the area of the ground truth segmentation, |*Y*| is the area of the semantic segmentation result, and 2|*X* ∩ *Y*| is the area of overlap (intersection) between |*X*| and |*Y*|.

To evaluate the performance of the algorithm in the detection and classification of NSCLC tumors, we calculated the sensitivity, specificity, and accuracy as shown in Eqs. 2, 3, and 4, respectively:


Eq. 2 $$Sen=\frac{TP}{TP+FN}\times 100$$

where *Sen* is the sensitivity, *TP* is the number of true-positive results (i.e., segmented slices in which a nodule was detected and classified as adenocarcinoma or SCC, subsequently being classified as adenocarcinoma or SCC on histology), and *FN* is the number of false-negative results (i.e., segmented slices in which a nodule was detected and classified as noncancerous, subsequently being classified as adenocarcinoma or SCC on histology).


Eq. 3 $$Spe=\frac{TN}{TN+FP}\times 100$$

where *Spe* is the specificity, *TN* is the number of true-negative results (i.e., segmented slices in which a nodule was detected and classified as noncancerous, subsequently being classified as noncancerous on histology) and *FP* is the number of false-positive results (i.e., segmented slices in which a nodule was detected and classified as cancerous, subsequently being classified as noncancerous on histology).


Eq. 4 $$Acc=\frac{TP+TN}{TP+TN+FP+FN}\times 100$$

where *Acc* is the accuracy, *TP* + *TN* is the total number of slices that were classified correctly, and *TP* +*TN* + *FP* + *FN* is the overall total number of slices evaluated.

### Training

The use of a GTX 1060 GPU accelerated the training process approximately 16-fold in comparison with training without a GPU. Two learning rates (10^−3^ and 10^−6^) were tested, and the best efficiency was achieved at the 10^−3^ rate. In this work, the adaptive moment estimation algorithm optimizer was also used, in order to minimize the cost function (cross-entropy) and maximize the DSC, being considered the optimizer that has been shown to converge faster. Adaptive moment estimation optimizer is an adaptive learning rate optimization algorithm specifically designed to train deep neural networks^**(^23^)**^. The learning runtime was about 12 h for batches of 8 or 18 images.

Initially, the 2D U-Net was pretrained from scratch by using 28,506 images from the TCIA NSCLC database with 40 epochs. After this initial learning step, a fine-tuning transfer learning process was applied to the 2D U-Net to improve the DSC performance. This fine-tuning learning step used an additional data set of 497 tumor-only images of 22 other patients from the TCIA NSCLC database. This extra learning step used an additional 20 epochs to improve the DSC performance of the 2D U-Net. We consider this adequate for detecting images with tumors without impairing the detection of true negatives, which account for the majority (77%) of the images.

### Segmentation

Semantic segmentation consists of separating an image into different regions and allocating each pixel to the part of the image to which it belongs. The NSCLC cases were detected by using the U-Net architecture. An additional 2,015 images containing NSCLC ground truth segmentation (17 different patient cases with NSCLC adenocarcinoma and SCC subtypes) were selected from the TCIA data set to validate the segmentation results.

**Figure 1:**
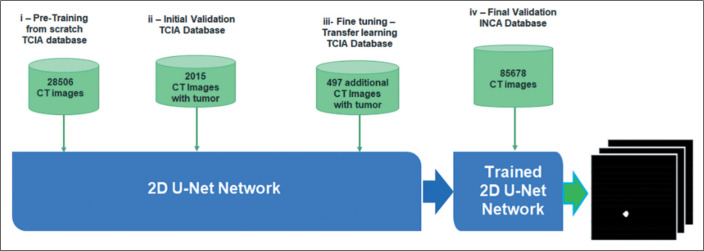
Flow chart of the script, from initial learning to the external validation test.

Figure 1 shows the steps in the semantic segmentation process, from the initial learning to the validation test: the 2D U-Net pretraining on the 28,506 images from the TCIA database; the initial validation test with segmentation metrics (DSC) related to the 2,015 images from the TCIA database; the fine-tuning step with 497 tumor-only images from the TCIA database to improve the segmentation performance of the 2D U-Net; and the final NSCLC validation test with 85,678 images from 104 INCA patients. The results were compared with the annotations available on patient electronic medical records to evaluate sensitivity.

### Detection

The NSCLC cases were detected by comparing the binary image outputs with the annotations available on patient electronic medical records to evaluate sensitivity.

### Classification

**Figure 2:**
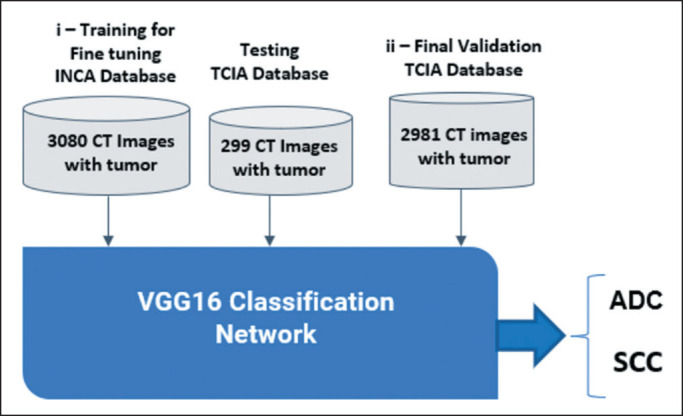
The training step (i) used 3,080 images from the INCA database, the testing step used 299 images from the TCIA database, and the validation step (ii) used 2,981 images from the TCIA database. ADC, adenocarcinoma.

To classify the NSCLC histologic subtype, we used the VGG16 architecture, with performance metrics including accuracy and the area under a receiver operating characteristic curve. Three learning rates (10^−7^, 10^−8^, and 10^−9^) were tested, and the best accuracy was achieved with the 10^−8^ rate. The process involved the following: fine-tuning training with 3,080 images from the INCA database and testing with 299 images from the TCIA database; validation using 2,981 images from the TCIA database; image preprocessing including centering, resizing to 112 × 112 pixels, and conversion to the red-green-blue color space; and data augmentation similar to what was used in the previous steps. Figure 2 illustrates the fine-tuning training with 3,080 images from the INCA database and the final validation with 2,891 images from the TCIA database.

## RESULTS

Table 1 shows TCIA and INCA epidemiological information from patients with each of the predominant histologic subtypes used in order to train the segmentation and detection DL algorithms. The pretraining step used 40 epochs, and the training process as a whole used 60 accumulated epochs.

**Table 1:** Histologic subtypes and ages of the patients used in training the DL model, by data set and gender.

Data set	Histologic subtype		Age (years) Mean ± SD
	Adenocarcinoma (n = 62) n (%)	SCC (n = 42) n (%)	
TCIA			
Gender			
Male	13 (61.9)	16 (43.2)	70.6 ± 9.4
Female	8 (38.1)	21 (56.8)	63.1 ± 9.1
INCA			
Gender			
Male	30 (48.4)	34 (81.0)	70.4 ± 7.9
Female	32 (51.6)	8 (19.0)	65.7 ± 8.7

SD, standard deviation.

**Figure 3:**
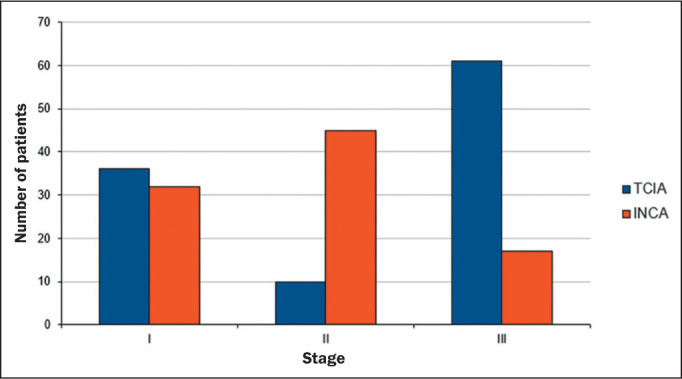
Staging of NSCLC in the TCIA and INCA data sets.

**Figure 4:**
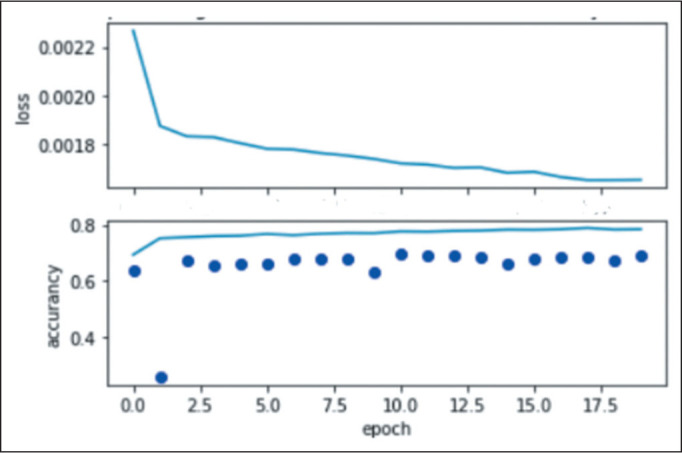
Performance of the proposed DL model (loss function and DSC segmentation accuracy of 0.68) during the 20-epoch fine-tuning step.

Figure 3 shows relevant clinical factors: the NSCLC patient staging for the early stage (stage I and stage II) and advanced stage (stage III) data sets. Comparing the INCA and TCIA data sets, we found that number of patients was similar for stage I but there were more INCA patients in stage II and more TCIA patients in stage III. The INCA data set had more patients in the early stages, whereas the distribution of patients was more homogeneous across the three stages in the TCIA data set. Figure 4 shows the DL model performance (loss function and DSC segmentation accuracy of 0.68) using the TCIA data set during the fine-tuning step to learn and test scripts of the first detection and segmentation process.

**Figure 5:**
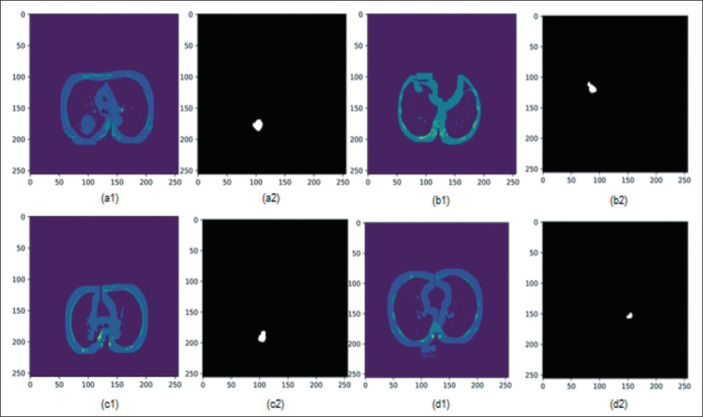
Examples of images of both lungs from four patients in the INCA data set, some with adenocarcinoma (**a,d**) and some with SCC (**b,c**). The lung-masked CT slices are shown in **a1**, **b1**, **c1**, and **d1**. The segmentation results are shown in **a2**, **b2**, **c2**, and **d2**.

Figure 5 shows the semantic segmentation result obtained and applied as input to the preprocessed image of the DL classification histological subtype step to four tumors, two classified as adenocarcinoma and two classified as SCC, located on both lungs. Each CT scan had an execution time of approximately 6 s to predict all 600 slices/patient.

For the test/validation image data set, the proposed DL model showed greater than 90% sensitivity and specificity for detecting both main histologic subtypes of NSCLC. After the fine-tuning step, the model was found to have a sensitivity of 91.7% and 90.4% for adenocarcinoma and SCC, respectively, with a specificity of 99.3% and 99.5%, respectively, and an accuracy of 84.5% and 89.6%, respectively, in INCA patients. The number of patients whose data were used in order to train the proposed DL model was balanced with the incidence rate by histological subtype (adenocarcinoma and SCC) and, as indicated above, the accuracy was 5% better for classifying SCC.

## DISCUSSION

During the training of the DL model proposed in our study, a DSC of 0.68 was achieved, compared with the 0.84 reported by Primakov et al.^**(^13^)**^, who did not differentiate among NSCLC subtypes. After fine-tuning, our model achieved a 99% specificity in tumor detection, which aligns with the results obtained by Lei et al.^**(^12^)**^ and indicates effective differentiation between images of tumors and non-tumors, with minimal false-positive results.

The TCIA data set used for training our model included 21 cases of adenocarcinoma, 37 cases of SCC, and 58 cases of other NSCLC subtypes, whereas the INCA data set use for external validation included 62 cases of adenocarcinoma and 42 cases of SCC. The predominance of SCC over adenocarcinoma in the TCIA data set constitutes a significant mismatch, given that adenocarcinoma is more prevalent in the population of Brazil^**(^2^,^30^)**^. To address this imbalance, the model should be fine-tuned with a more balanced data set, ideally with twice as many cases of adenocarcinoma as cases of SCC. Such adjustments are crucial for increasing accuracy and addressing population-specific biases^**(^25^,^26^)**^.

The present study utilized adequate preprocessing with a lung mask, allowing satisfactory results to be obtained within 60 epochs, much fewer than the 100–500 epochs required in similar studies^**(^24^)**^. That level of efficiency was due to improved preprocessing and training techniques. The proposed method also demonstrated good agreement with physician reports on electronic medical records for INCA patients, in keeping with the results obtained by Lei et al.^**(^12^)**^ and Paing et al.^**(^14^)**^.

For identifying adenocarcinoma and SCC, our detection algorithm achieved an accuracy comparable to that reported by Aydın et al.^**(^27^)**^, with a sensitivity similar to that reported by Primakov et al.^**(^13^)**^. The results of the present study, distinguishing between the main histologic subtypes, aligns well with the findings of nondichotomous classification studies such as those conducted by Chaunzwa et al.^**(^28^)**^ and Pang et al.^**(^29^)**^. After testing various image sizes, we chose 224 × 224 pixels, which is suitable for the VGG16 network input, the same input as the original network architecture.

Overall, increasing accuracy in classifying NSCLC histology subtypes remains challenging, particularly for multiple subtypes, due to the need for larger and more diverse data sets. Classification accuracy tends to improve with a larger number of cases for each subtype in the data set.

Our study has some limitations. First, the proposed DL model was not trained to make an initial diagnosis of cancer, instead being trained and tested on patients already diagnosed with neoplasia, with the aim of differentiating between histologic subtypes. In addition, the model was tested on data from a single scanner. Although that might be considered a limitation, it highlights the feasibility of applying DL for histologic differentiation in this controlled setting.

Upregulated expression of programmed death-ligand 1 protein is important for some immunotherapies. Therefore, future studies could improve upon our research considerably by incorporating genomics.

We believe that our findings could facilitate the development of advanced diagnostic tools to improve health care, mainly in regions with limited access to medical specialists. Another area in which our model could be useful is in the diagnosis of lung cancer due to electronic cigarette use, the incidence of which has been increasing, particularly among young people.

## CONCLUSION

This work offers a way to classify the main histologic subtypes of NSCLC in a specific population, with a DSC similar to or better than that obtained in previous studies of this topic. This approach could be useful in the automated classification of lung cancer subtypes in local populations, using DL networks trained on public image data sets. The use of the DL model proposed here could also help oncological radiologists in image analysis and processing.
